# Selections

**Published:** 1894-02

**Authors:** 


### Selections.

### FORMALINE: AN IDEAL HARMLESS GERMICIDE.

Formaldehyde (HCOH) as known is a gas under normal conditions of temperature and pressure; it is readily soluble in water, and such a solution of forty-per-cent. strength is put forward under the name of "formalin." This liquid mixes in all proportions with water, and any required dilution can be readily prepared; thus, for instance, by mixing 1 part of formalin with 40 parts of water, 41 parts of a one-per-cent. solution are at once obtained.

Even at ordinary temperatures, formalin gives off gaseous formic aldehyde and the evolution of gas is of course accelerated by application of heat.

Exhaustive and numerous experiments on the bactericidal power of formalin were carried out by Dr. J. Stahl. Berlioz and Frillat, already mentioned, had found that anthrax bacilli were killed by a dilution of 1 : 50,000, while Aronson stated that solutions of 1 : 20,000 prevented the development of typhus and anthrax bacilli as well as of *staphylococcus pyogenes aureus.* Stahl's observations proved that, after one hour's exposure to a one-per-mille, or a quarter of an hour's exposure to a one and-one-third-per-mille solution of formalin, the most resistant forms of micro-organism were destroyed. At the least, therefore, formalin is equal in germicidal power to sublimate, and under certain conditions would be superior where albuminoid solutions are concerned.

As already intimated, great importance is attached to the applicability of the ideal antiseptic in gaseous or vaporous form, as only in this way can we conveniently disinfect large rooms and more delicate articles in closed apparatus. The experiments carried out by Dr. Stahl with formalin vapor were made in a large glass bell in which was set a small table carrying potatoes freshly inoculated with pure cultures of typhus, anthrax, cholera, etc. The glass bell stood upon an iron plate which exactly closed it. The experiments showed that 2.5 volume per cent. of formalin in the air destroyed all micro-organisms in a quarter of an hour. Undoubtedly even better results would have been obtained could a constant stream of formalin vapor have been conducted over the cultures.

Summing up the results of experiments, the properties of formalin may be expressed as follows:

1. It has an extraordinarily active microbicide power similar to that of sublimate.

2. It is comparatively non-poisonous.

3. It attacks only the substance of the contagious material, leaving intact the articles treated, whether of organic or inorganic nature.

4. It is very readily employed under all circumstances either as liquid or in gaseous form.

It is a marked advantage of the vapor of formalin that its specific gravity closely approximates to that of the air, so that there is no difficulty in keeping the atmosphere of an enclosed space uniformly impregnated with formalin vapor.

Dr. Stahl recapitulates the methods of using formalin by spray and vaporization, pointing out that, for the superficial disinfection of furniture, articles of clothing, etc., spraying with a one-half-per-cent. solution should be resorted to and carried out with as much energy as possible. In gaseous form the preparation promises to do good service in the dry disinfection of more delicate articles in closed vessels as well as of furs, dressing material, and the like.—*Notes on New Remedies.*

---

### RESECTION OF THE INFERIOR MAXILLA WITH IMMEDIATE PROSTHESIS OF AN ARTIFICIAL JAW.

*Dr. P. Michaux.*—I wish to communicate to the Society the remarkable results obtained by me in a case of immediate prosthesis after complete excision of the right half of the inferior maxilla, by the method recommended by Dr. Claude Martin (Lyons).

The case is briefly as follows: epithelioma of the lower lip of four or five years' duration; patient aged thirty-six; removal by free excision on June 27. Recurrence on September 15, and rapid invasion by the disease of the body of the lower jaw on the right side. On November 2, the right half of the inferior maxilla was excised, and an artificial jaw inserted at one and the same operation. The artificial maxilla employed was made of vulcanized india-rubber with a number of perforations according to Dr. Martin's suggestions (a central tube with a buccal orifice, and communicating with a whole series of channels which open all along the free edge of the body and ramus of the artificial jaw, so as to permit of the free irrigation of the region). It was screwed by means of two metallic plates to the internal surface of the remaining half of the inferior maxilla, while it was also connected by a spring with another apparatus fixed to the under surface of the superior maxilla.

When the patient recovered consciousness the action of the apparatus proved very satisfactory. The external wound was dressed with iodoform, while the interior of the mouth was constantly irrigated through the perforations in the artificial jaw. This method of irrigation is perfect; the patient's temperature never rose above the normal. At the end of seven days the parts had completely healed, and the sutures were removed. This is the twelfth day after the operation and the inferior maxilla already possesses an extensive range of movements. The patient takes soup without the slightest difficulty, and he speaks tolerably well. In short, the results of the operation are most satisfactory, especially in presence of the unsightly deformity which is produced by resection of half of the lower jaw by the ordinary methods, such as want of concordance of the teeth, deviation of the remaining half of the maxilla, difficulty of speech, mastication, etc. There was a total absence of the grave septicæmic and infectious complications which are so frequent after this operation, a result which is probably due to the method of irrigation through the perforations in the artificial jaw.

I consider, therefore, that the prosthetic apparatus devised by Dr. Martin is a distinct advance in the surgery of the jaw. It gives better results than those obtainable with a secondary operation (always a difficult procedure) or the simpler appliances employed in Germany by Bœnecken.

*Dr. Prengrueber.*—About three months ago I performed an operation similar in every respect to that just described by Dr. Michaux, on the indications furnished by Dr. Cl. Martin. It was a case of cancer of the floor of the mouth involving the inferior maxilla and the anterior half of the tongue. A portion of the body of the jaw on either side of the symphysis and the anterior half of the tongue having been exeised, the immediate insertion of an apparatus proved especially useful in enabling the tongue to be at once fixed to the apparatus. The operation gave excellent results, but, unfortunately, the disease recurred at the end of a month.—*The Med. Week*, Paris.

